# What influences graduate medical students’ beliefs of lower back pain? A mixed methods cross sectional study

**DOI:** 10.1186/s12909-022-03692-1

**Published:** 2022-08-20

**Authors:** John G. K. Inman, David R. Ellard

**Affiliations:** 1 Warwick Medical School, Medical School Building, University of Warwick, Coventry, CV4 7HL UK; 2 Warwick Clinical Trials Unit, University of Warwick, Coventry, CV4 7HL UK

**Keywords:** Back pain, Medical students, Healthcare students, Education, Health beliefs

## Abstract

**Background:**

Low back pain (LBP) is a common condition with substantial associated disability and costs, best understood using a biopsychosocial approach. Research demonstrates that beliefs about LBP are important, with biomedical beliefs influencing practitioner’s management and patient recovery. Beliefs about LBP can be inconsistent amongst healthcare and medical students. The aim of this study was to investigate graduate medical student’s beliefs of LBP and what influences them.

**Method:**

A cross sectional mixed methods study of Phase 1 (first year) and Phase 3 (third and fourth year) current graduate medical students at the University of Warwick (MBChB) was conducted. Participants were recruited via voluntary response sampling. A survey investigated LBP beliefs, utilising the Back Beliefs Questionnaire (BBQ) and Health Care Providers’ Pain and Impairment Relationship Scale (HC-PAIRS). Qualitative data was collected on what influences beliefs about the causes and management of LBP, which was analysed descriptively using thematic analysis.

**Results:**

Fifty-seven students completed the questionnaire (61% female), with a mean age of 27.2 years. Eighty two percent of participants reported a history of LBP. Median BBQ scores were 31.5 for phase 1 and 31 for phase 3, with median HC-PAIRS scores of 57 and 60 for phase 1 and phase 3 students respectively. Three main themes emerged from the qualitative data: Sources of influence, influence of personal experience and influence of medical education. Participants discussed single or multiple sources influencing their beliefs about the causes and management of LBP. Another main theme was the influence of experiencing LBP personally or through discussions with family, friends and patients. The final main theme described the influence of medical education, including lectures, seminars and clinical placements.

**Conclusions:**

The HC-PAIRS and BBQ scores suggest graduate medical students in this sample tended to have positive beliefs about the outcome of LBP and functional expectations of chronic LBP patients, consistent with other healthcare students. The findings from qualitative data suggest how medical students form beliefs about the causes and management of LBP is complex.

**Supplementary Information:**

The online version contains supplementary material available at 10.1186/s12909-022-03692-1.

## Background

Low back pain (LBP) is a common medical condition that poses a substantial healthcare challenge due to its high prevalence, associated disability and financial cost. It is estimated up to 80% of people will experience an episode of LBP during their lifetime [1]. LBP is in the top ten causes of ‘Disability Adjusted Life Years’ and has a prevalence of 7.6% predicted to increase [2]. As a result, management incurs considerable costs, which in the United Kingdom (UK) are estimated at £12 billion [3].

Around 90% of LBP is ‘non-specific’, where pain located between the twelfth rib and gluteal folds is not attributable to an identifiable cause [4]. It is recognised as a complex condition with multiple underlying and interacting mechanisms, best understood with a biopsychosocial approach [5, 6]. This diagnostic model theorises a disease presentation as having biological, psychological and social components, therefore capturing the multiple interactions leading to LBP [7, 8]. For example, the relations of local anatomical and nervous system changes, psychological factors such as anxiety and kinesiophobia, in addition to social aspects such as work absence [8–11]. Due to its complexity, management of LBP can be difficult; treatments such as analgesia, exercise and manual therapy have modest treatment effects [12]. Clinical guidelines have been published to provide clarity on best practice, with a broad consensus of recommendations internationally [13]. In the UK, these include assessing psychological factors for disability, with recommendations including psychological therapies, education and self-management advice [14].

Despite widespread use of guidelines, management of LBP is suboptimal due to a ‘translation gap’ where practice does not reflect research [12, 15, 16]. Alongside biopsychosocial approaches to LBP, biomedical conceptions focusing on identifying specific anatomy causing pain remain pervasive in healthcare professions [5, 12, 17, 18]. Whilst patently crucial for identifying serious pathology, practitioners with biomedical approaches to LBP management have been shown to provide advice inconsistent with guidelines, including routine use of imaging and recommending avoidance of activity [19–22]. Furthermore, the diversity of treatments for LBP means management can be interdisciplinary involving multiple practitioners [14]. Studies undertaken by Briggs et al. and Kennedy et al. investigating healthcare students’ beliefs of LBP demonstrated there was also lack of consistency between physiotherapy, chiropractic, nursing and medical students [15, 23]. Moreover, this was found when considering persistent pain and judging harmfulness of daily activities [24, 25]. Incongruent management can lead to contrasting advice and investigations between individuals and healthcare professions, undermining patients wanting clear and consistent explanations for LBP [18, 22, 23, 26, 27]. Addressing conflicting understanding between healthcare professionals (HCPs) and students regarding LBP and disability is important to improve care and narrow the ‘translation gap’.

Managing psychological aspects of LBP, including mental health symptoms such as anxiety as well as cognitions and beliefs about LBP, is important because they are risk factors and predictors of pain chronicity [20, 28–30]. An individual’s belief of what is causing their LBP can affect their emotional response and in turn their behaviour, which can affect disability [31]. Biomedical conceptions of LBP may be associated with the development of ‘fear avoidance’ and ‘negative’ beliefs, that activity can lead to further injury and exacerbation of pain, triggering avoidance of activity and increasing the likelihood of chronicity [28, 30, 32, 33]. HCPs are the most pervasive source of biomedical understanding for patients and it could be proposed have strongest influence over patients’ LBP beliefs [20, 34]. Whilst it cannot be ascertained if biomedical beliefs of LBP cause increased disability or not, there is an important relationship between the two [6, 35]. However, despite suggestions that biomedical beliefs can lead to increased disability, research has demonstrated better outcomes in patients with LBP treated by physiotherapists with biomedical beliefs [36].

Given evidence for inconsistencies of LBP beliefs amongst HCPs and students, one could suggest it is important shared understanding about the causes and management of LBP is fostered during training to reduce future disparity between research and practice. A scoping review by Lewis and Battglia exposed suboptimal understanding of psychosocial factors implicated in LBP amongst health science students [37]. In addition, graduate entry medical students displayed more ‘fear avoidant’ beliefs about LBP in relation to daily physical activity when compared to physiotherapy students [23]. Negative views regarding chronic pain have also been found in medical students [15]. Despite these findings, beliefs and attitudes towards pain and LBP amongst healthcare and medical students have been shown to improve during study, demonstrating the effectiveness of education [24, 38, 39]. For example, medical students have been found to believe LBP myths including ‘back pain is likely to be caused by heavy lifting’. However, following a seminar this was significantly dispelled [40]. There is further evidence for using other specific educational interventions to address unhelpful beliefs, such as e-learning modules [41, 42].

Research discussing influences over medical students’ beliefs of LBP using quantitative measures has been inconclusive. For example, a personal history of LBP was not associated with a change in BBQ or HC-PAIRS scores of physiotherapy and healthcare students [39, 43]. However, in another study of female healthcare students using the BBQ and HC-PAIRS, participants with a history of LBP demonstrated more negative beliefs of LBP [44]. Collecting qualitative data may help to further understanding of what influences medical students’ beliefs of LBP.

The aim of this study was to investigate graduate entry medical students’ beliefs of LBP and any characteristics associated with beliefs. Qualitative data was gathered to explore what influences students’ beliefs about the causes and management of LBP.

## Methods

### Study design

A cross sectional mixed methods anonymous survey was used to investigate beliefs about LBP. Ethical approval was granted by the University of Warwick’s Biomedical and Scientific Research Ethics Committee (BS-REC). Informed consent was obtained from all participants before taking part.

### Participants

Participants were students from the University of Warwick’s four-year graduate entry medical degree (MB ChB). All students on this course hold previous university qualifications, including healthcare degrees. MBChB students in ‘Phase 1’ of the course is made up of students in their first year, with students entering ‘phase 3’ in their third and fourth year of study.

### Data collection

#### The survey

Informed consent was obtained from all participants before beginning the survey. The survey collected demographic information in addition to quantitative and qualitative data. Demographic characteristics included gender, age, previous degree of study and LBP history. Beliefs of LBP were examined using the Back Beliefs Questionnaire (BBQ) and Health Care Providers’ Pain and Impairment Relationship Scale (HC-PAIRS). The BBQ evaluates beliefs regarding the inevitable outcome of LBP and has been validated for use in the general population [45, 46]. The HC-PAIRS examines HCPs expectations for functional impairment in chronic LBP patients [47]. The BBQ and HC-PAIRS have good internal consistency for assessing LBP beliefs (Cronbach’s alpha of 0.87 and 0.78 respectively) and have been used in comparable research assessing healthcare students’ LBP beliefs [15, 23, 24, 39, 47–49]. Qualitative data was gathered through open text response boxes for two questions investigating what students thought had influenced their beliefs about the causes and management of LBP. The survey was hosted on ‘Qualtrics’ software and pilot tested by four independent students for ease of completion. An example of the survey can be found in Additional file 1.

#### Procedure

A hyperlink to the survey was distributed via email, created with ‘Qualtrics’ software. Access was provided for four weeks starting 29th November 2021.

#### Data analysis

The HC-PAIRS consists of fifteen statements regarding chronic LBP and impairment, scored on a seven-point Likert scale. The scores are added together, with items one, six and fourteen inverted. A lower score suggests positive beliefs and attitudes that pain complaints do not justify impairments and disability. The BBQ uses fourteen statements about LBP with responses logged on a five-point Likert scale, containing five statements acting as distractors. To calculate the total, the scores are reversed and the nine question responses added together, with a lower score indicating more negative beliefs. Given the small sample size, appropriate descriptive statistics were generated to display the data.

Qualitative data gathered from text responses was analysed with thematic analysis [50, 51]. Responses to the open questions were read repeatedly to gain familiarity with the data. Phrases were assigned a descriptive code, with an inductive approach taken to allow codes to emerge from what participants said. Themes capturing meaningful patterns in relation to the research question were formed from these codes, which were reviewed constantly during analysis to ensure they were an accurate representation of the data.

## Results

### Demographic characteristics

A total of 57/577 (9.8%) students invited completed the questionnaire, with 12/204 (5.8%) of Phase 1 students and 45/373 (12%) of Phase 3 students. Incomplete questionnaires were included in the final analysis if they had complete scores for either of the questionnaires or responses to the qualitative data questions. There were more responses from females (61%), with 82% of participants reporting previous history of LBP. Responses to the open questions were made in 56 (98%) of finished questionnaires. The mean age of respondents was 27.2 years. Further characteristics are displayed in Tables 1 and 2.

**Table 1:** Demographic profile of participants

	*N* = 57 n (*%*)	^a^Phase 1 Students *n* = 12 n (%)	^b^Phase 3 students *n* = 45 n (%)
Gender			
Male	22 *(39)*	4 *(7)*	18 *(32)*
Female	35 *(61)*	8 *(14)*	27 *(47)*
Age			
21–25	22 *(39)*	6 *(10)*	16 *(28)*
26–30	27 *(47)*	5 *(8)*	22 *(38)*
31–35	3 *(5)*	1 *(2)*	2 *(4)*
36–40	4 *(7)*	0 *(0)*	4 *(7)*
> 41	1 *(2)*	0 *(0)*	1 *(2)*
Have you previously experienced lower back pain?			
Yes	47 *(82)*	11 *(19)*	36 *(63)*
No	10 *(17)*	1 *(2)*	9 *(15)*
Are you currently experiencing lower back pain			
Yes	10 *(17)*	1 *(2)*	9 *(15)*
No	47 *(82)*	11 *(19)*	36 *(63)*
Previous degree			
Biomedical Science	11 *(19)*	2 *(5)*	9 *(16)*
Psychology	8 *(14)*	2 *(5)*	6 *(10)*
Biology	3 *(5)*	1 *(2)*	2 *(5)*
Physiotherapy	3 *(5)*	2 *(5)*	1 *(2)*
Biochemistry	2 *(5)*	0 *(0)*	2 *(5)*
Neuroscience	2 *(5)*	0 *(0)*	2 *(5)*
Pharmacology	2 *(5)*	0 *(0)*	2 *(5)*
Radiography	2 *(5)*	0 *(0)*	2 *(5)*
Pharmacy	2 *(5)*	0 *(0)*	2 *(5)*
Music	2 *(5)*	1 *(2)*	1 *(2)*
Sports and Exercise Science	2 *(5)*	2 *(5)*	0 *(0)*
Physiology	1 *(2)*	0 *(0)*	1 *(2)*
Mathematics	1 *(2)*	1 *(2)*	0 *(0)*
Accounting and Finance	1 *(2)*	0 *(0)*	1 *(2)*
Geophysics	1 *(2)*	0 *(0)*	1 *(2)*
Medical Physiology	1 *(2)*	0 *(0)*	1 *(2)*
Natural Science	1 *(2)*	0 *(0)*	1 *(2)*
Biological Science	1 *(2)*	0 *(0)*	1 *(2)*
Physiology	1 *(2)*	0 *(0)*	1 *(2)*
Chemical engineering	1 *(2)*	0 *(0)*	1 *(2)*
Clinical physiology	1 *(2)*	1 *(2)*	0 *(0)*
Engineering	1 *(2)*	0 *(0)*	1 *(2)*
Osteopathy	1 *(2)*	0 *(0)*	1 *(2)*
Paramedic Science	1 *(2)*	0 *(0)*	1 *(2)*
English	1 *(2)*	0 *(0)*	1 *(2)*
Philosophy	1 *(2)*	0 *(0)*	1 *(2)*
Politics and French	1 *(2)*	0 *(0)*	1 *(2)*
Law	1 *(2)*	0 *(0)*	1 *(2)*
Physiological sciences	1 *(2)*	0 *(0)*	1 *(2)*

^a^MBChB Phase 1 = First year graduate entry medical students

^b^MBChB Phase 3 = Third- & fourth-year graduate entry medical students

**Table 2:** Questionnaire responses

Questionnaire responses	Total *n* = 57	^a^Phase 1 Students *n* = 12	^b^Phase 3 students *n* = 45
Back Beliefs Questionnaire Score			
Range	22–41	27–41	22–40
Mean (Standard Deviation)	31.8 (4.7)	32.6 (4.6)	31.6 (4.8)
Median	31	31.5	31
Lower Quartile	28	29.5	28
Upper Quartile	35	35.5	35
Interquartile Range	7	6	7
Health Care Providers’ Pain and Impairment Relationship Scale (HC-PAIRS)			
Range	21–81	43–81	45–78
Mean (Standard Deviation)	58 (9.9)	57.5 (11.2)	59.4 (9.7)
Median	60	57	60
Lower Quartile	53.75	48.5	55
Upper Quartile	65	63.5	65
Interquartile Range	11.25	15	10

^a^MBChB Phase 1 = First year graduate entry medical students

^b^MBChB Phase 1 = Third- & fourth-year graduate entry medical students

### BBQ and HC-PAIRS scores

**Fig. 1:**
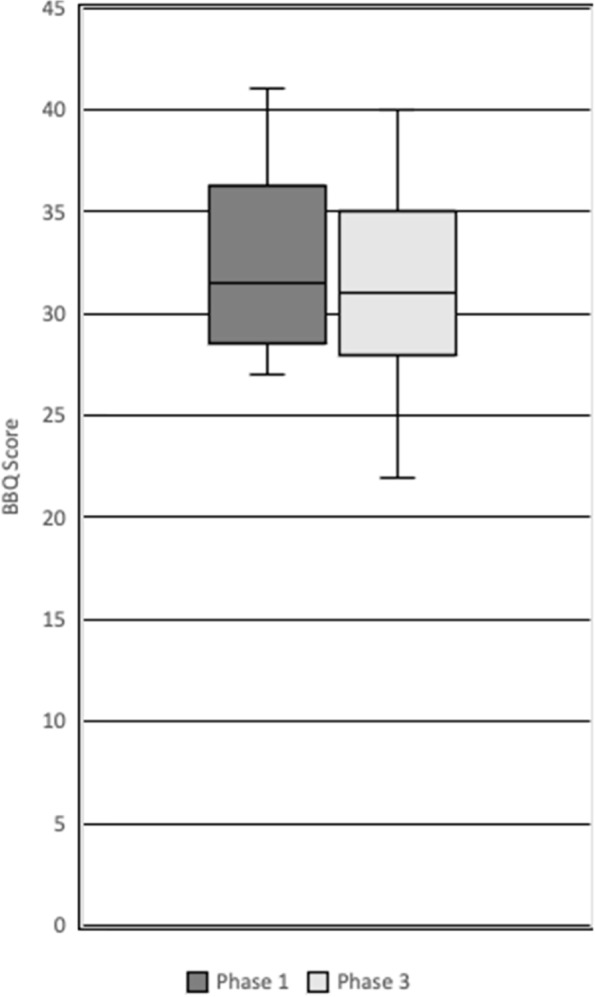
Back Beliefs Questionnaire (BBQ) Scores of Phase 1 and 3 Students

Figure 1 shows the distribution of BBQ scores between phase 1 and phase 3 students. The median scores for both groups are almost equal, with a values of 31.5 and 31 for phase 1 and 3 respectively. Additionally, there is little variability of the interquartile range for both year groups. However, the spread of BBQ scores is more variable amongst phase 3 students. Scores higher than 27 indicate disagreement with beliefs about the negative consequences of LBP, suggesting this sample of medical students tended to have positive beliefs about LBP [35].

**Fig. 2:**
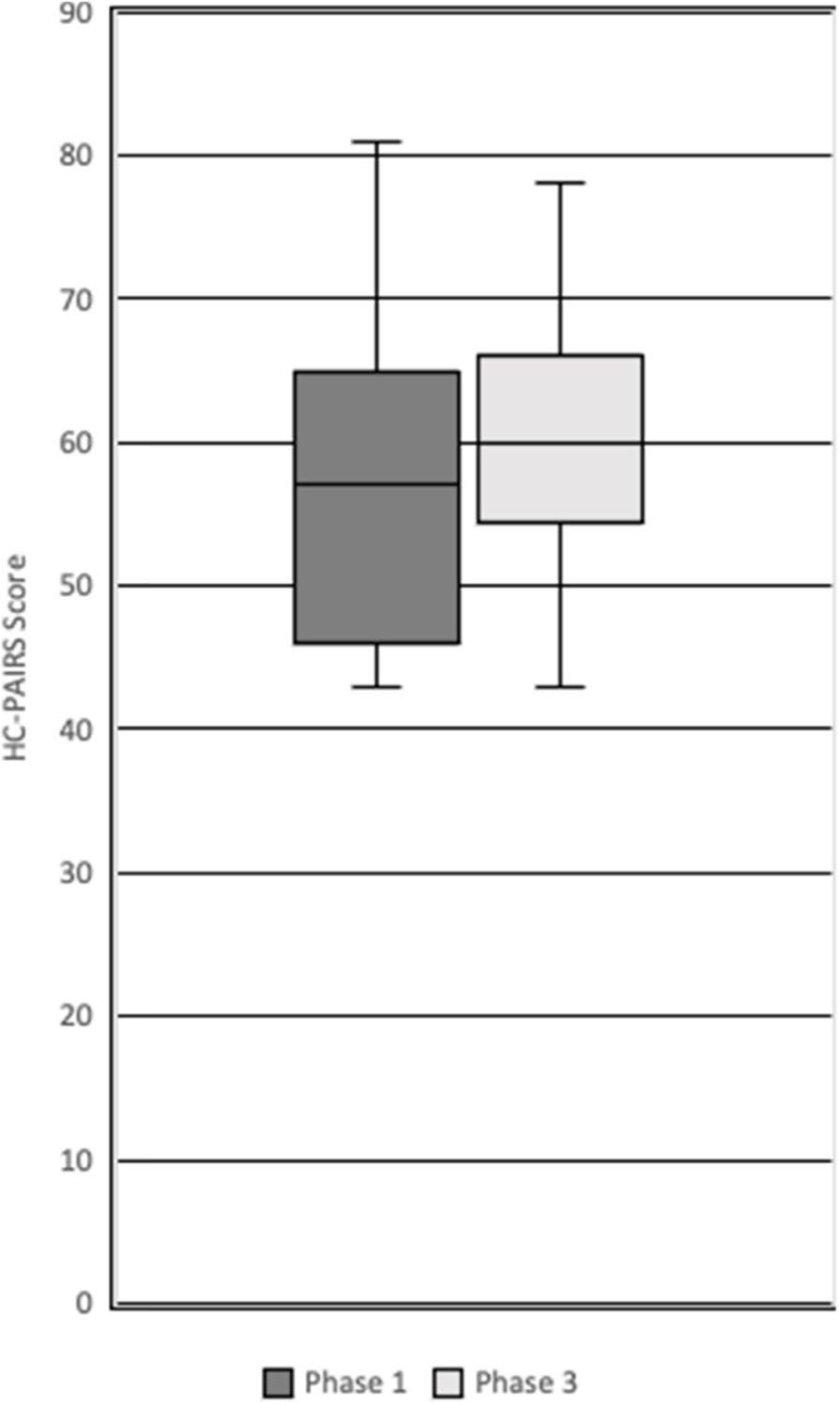
Healthcare Providers Pain and Impairment Relationship Scale (HC-PAIRS) Scores of Phase 1 and 3 Students

The distribution of HC-PAIRS scores is displayed in Fig. 2. As with the BBQ scores, the median is similar between the 2 year groups, with scores of 57 in phase 1 and 60 in phase 3. There.

is increased variability in the distribution of the interquartile range, with a lower quartile score in phase 1 compared with phase 2. The overall spread of scores is similar across both student groups.

### Qualitative results

Participants were invited to respond to two questions, “What has influenced your beliefs about the causes of low back pain?” and “What has influenced your beliefs about the management of low back pain?”, with encouragement to provide as much detail as possible in an open text box. All responses were transcribed verbatim into a Word document for analysis. Responses varied from a couple of words to multiple sentences. This survey method of qualitative data collection compared to interviews is restrictive for interpretive analysis as responses represented a ‘snapshot’ of a participant’s evaluation of the question with no opportunity to explore or clarify participants perceptions and meaning, which would be possible in an interview. However, using an inductive and descriptive approach to analysis, meaningful themes in relation to the research question were derived. The lead researcher JI, a male third year medical student with 4 years of qualitative research experience, conducted the analysis and discussed the final themes with DE and a senior back pain researcher. A table with detailed supporting quotes is located in Additional file 2.

Three main themes emerged:

1. Sources of influence
2. Influence of personal experience
3. Influence of medical education

#### Sources of influence

Sources of influence relates to how many sources participants described as influencing their beliefs about the cause and management of LBP. Some participants referenced multiple sources, across their personal and university learning experiences. In contrast, other participants responded with only one source. Exemplar quotes are shown in Table 3.

**Table 3:** Sources of influence

Sub-theme	*Quote [Participant ID]*
Single source	*“My experience of lower back pain and how it affected my work life and hobbies” [2.5]* *“Seeing family members with lower back pain” [2.6]*
Multiple sources	*“Med school education, physiotherapists, chiropractor, GP (General Practitioner), family, friends” [3.9]* *“Personal experience and that of friends and family (anecdotal) Phase 2 [second year] lectures on Lower Back Pain and MSK drugs GP Placement - seeing patients with the GPs and first contact physio” [4.0]*

#### Influence of personal experience

The second main theme relates to the personal experience of LBP as influencing beliefs about both the cause and management of LBP. This theme was consistent in responses from participants in both year groups but particularly in phase 1 students. This can be split into three sub themes, with example quotes shown in Table 4.

**Table 4:** Influence of personal experience

Sub-theme	*Quote [Participant ID]*
2.1 Individual’s personal experience with LBP	*“Personal experience - I have got lower back pain from driving long distances from work and sitting down a lot from work” [3.2]* *“In my personal experience I have had osteopathy on my lower back and found that to help..” [3.5]*
2.2 Personal experience of others with LBP	*“When struggling with pain, my Mum often reported frequent movement to help..” [2.8]* *“Something that has really stuck with me was talking to a patient with a long term back issue who said they regret getting back surgery for it” [3.2]*
2.3 Clinical personal experience of LBP	*“I also had exposure assessing many lower back pain patients and seeing the different causes” [2.1]* *“Knowledge & experience of supporting patients as a physiotherapist both with acute & chronic lower back pain” [1.1]*

##### Individual’s personal experience with LBP

Participants described their own personal experience having LBP, what they believed caused it and experience of management, as influencing their own beliefs. In response to the question about the cause of LBP, some references to disability were made. When responding to the question about LBP management, comments on perceived success of management received were made.

##### Personal experience of others with LBP

This describes participants referencing the influence of the personal experience of LBP by others such as their friends, family or patients.

##### Personal experience of LBP in a clinical context

Participants from a healthcare background described their clinical experience encountering patients with LBP. Students with experience of professions with musculoskeletal training (Physiotherapy and Osteopathy) were less focused on their current medical training in responses.

Comments were also made about the use of social media to access the opinions of HCPs and others about LBP. Participants additionally referenced previous education covering LBP as influencing their beliefs. Descriptive quotes are shown in Table 5.

**Table 5:** Influence of personal experience

Sub-theme	*Quote [Participant ID]*
Influence of social media Influence of previous degree	*Other things include social media professionals such as physiotherapists and chiropractors who show exercises to help manage lower back pain. [1.8]* *“..the basis for most of my understanding about different types of pain management has stemmed from my Neuropsychiatry studies..” [3.7]*

#### Influence of medical education

This theme describes the influence of participants current medical training over their beliefs, with references to learning experiences related to the course. Students in the sample from phase 1 did not respond with any reference to medical training. This can be split into three sub themes, with supporting quotes displayed in Table 6.

**Table 6:** Influence of medical education

Sub-theme	*Quote [Participant ID]*
3.1 From university	*“A lecturer on our course explained how difficult lower back pain can be to manage due to the interplay between physical and psychological components.” [5.5]* *“I think lectures on the management of lower back pain in Phase II [second year] have influenced my beliefs the most” [5.3]”*
3.2 From self-study	*“..remaining knowledge I would have got from studying, why for me mainly comprises of online resources - Zeros to Finals, YouTube videos.” [4.8]* *“Learning through.. BMJ Best Practice and NICE (*National Institute for Health and Care Excellence) *guidelines” [6.3]*
3.3. From clinical placements	*“I found GP placements to be the most useful when learning about low back pain as it is a common presenting complaint” [5.7]* *“Discussions with GPs when in their practice and meeting with patients with low back pain.” [4.6]*

##### From university

Beliefs were influenced by university learning opportunities, such as lectures, seminars and discussions with teaching staff.

##### From self-study

Beliefs were influenced in some by self-directed learning, accessing formal sources of information such as peer reviewed literature.

##### From clinical placements

Respondents described the influence of observing HCPs on clinical placements, mentioning General Practitioners (GP) and physiotherapists.

### Minor themes

A minor theme that emerged described uncertainty when dealing with LBP, in relation to it causes and management. Participants described the difficulty and challenge of managing LBP from the uncertain prognosis and variable effectiveness of treatments. In addition, students who were HCPs mentioned the influence of their previous studies and professional role suggesting this had an influence on beliefs. Quotes supporting these minor themes are displayed in Table 7.

**Table 7:** Minor themes

Sub-theme	*Quote [Participant ID]*
Uncertainty around LBP	*“more often than not, lower back pain seems to be a chronic issue without any confirmed aetiology.” [4.3]* *“The management seems more about relieving the symptoms/pain than progressive improvement.” [1.9]* *“my perception is these interventions are not effective for everyone and many people simply live with near constant lower back pain which is exacerbated at times.”* [3] *“it seems like it’s a balance of pain medication, and perhaps physiotherapy exercises, with not much else of use.” [5.6]*
Influence of previous degree	*“Previous undergraduate education in osteopathy... I also had exposure to assessing many lower back pain patients and seeing the different causes [2.1]* *“Knowledge & experience of supporting patients as a physiotherapist”[1.1]* *“Previous degree as a physiotherapist” [2.3]* *“the basis for most of my understanding about different types of pain management has stemmed from my Neuropsychiatry studies” [3.7]*

## Discussion

This study set out to investigate medical students’ beliefs of lower back pain. Although this is a small sample, scores are comparable with research using the BBQ and HC-PAIRS questionnaire to examine medical student’s beliefs of LBP. Moreover, the qualitative data collected offers a descriptive insight into the multiple influences affecting beliefs in this sample of graduate medical students. These emerging themes contribute to the understanding of what influences healthcare students’ beliefs of LBP, given the inconsistency of quantitative findings in the literature, which may be a valuable area to explore in future research.

The results from both questionnaires suggesting that medical students tend towards positive beliefs about the consequences of LBP, as well as functional expectations of chronic LBP patients, is consistent with previous research of medical and physiotherapy students. For example, mean BBQ scores for Phase 3 students are similar to studies of medical students conducted by Kennedy et al. and Briggs et al. with mean scores of 31.0 and 32.6 respectively [15, 23]. This also indicates participants have more positive beliefs in comparison to the general population, with a systematic review by Morton et al. describing 8 out of 12 studies having mean BBQ scores of less than 27 that suggests negative beliefs about the inevitable outcome of LBP [35]. Additionally, mean HC-PAIRS scores were comparable to a study of final year medical and physiotherapy students, suggesting positive attitudes towards pain and functioning [24, 39, 52]. For example, Ryan et al. and Augeard et al. found their samples of final year physiotherapy students to have mean HC-PAIRS scores of 57.4 and 55.6 [24, 52]. Mean scores of final year medical students in a study conducted by Morris et al. were 56.4 [39]. The number of students reporting a history of LBP (82%) is comparable to epidemiological data of the general population and slightly higher than findings of 72.1 and 73.4% in other samples of medical students. This could be due to the average age (27.2 years) increasing exposure to LBP [1, 53, 54].

One consistent theme was the variety of sources described as influencing beliefs, suggesting how medical students form beliefs is complex. Whilst some students discussed only one, such as their own experience of LBP or medical training, others mentioned multiple. Personal experience was one of the overarching themes to emerge, with students in both phases citing their own experience of LBP as influencing their beliefs about causes and management. This suggests experiencing LBP influences student’s beliefs, although understanding how requires further exploration. Research assessing healthcare students’ beliefs in relation to previous LBP experience using quantitative measures is conflicting. For example, there was no difference in HC-PAIRS scores of first and final year medical students with current or a history of LBP in research assessing attitudes towards chronic LBP [39]. Moreover, there was no association between a history of chronic LBP and changes to HC-PAIRS scores in Australian and Brazilian physiotherapy students [43]. A lack of change was also found in the BBQ scores of third year healthcare students with a history of LBP, although it did lead changes in scores measuring fear avoidance [23]. In contrast, female undergraduate healthcare students without a history of LBP demonstrated more negative beliefs, using questionnaires including the HC-PAIRS and BBQ [44]. Additionally, other aspects of personal experience may need to be considered, such as the influence of the experience of other individuals with LBP. This was evident in the comments of multiple students, describing the experience of family members, friends and patients as influencing their own beliefs.

There was a wide range of subjects previously studied by students on this graduate course, with forty-two having degrees in science, engineering and maths. Some mentioned the influence of their previous degree over their LBP beliefs, such as students with neuroscience giving examples of their understanding of pain neuroscience. For previous HCPs, this came from exposure to LBP in clinical encounters and previous university teaching, with description of their previous profession in their response. This may suggest an enduring influence of professional identity and prior knowledge on students from healthcare backgrounds who have encountered LBP, such as physiotherapy [55, 56].

Some responses described accessing professional and non-professional opinions on LBP via social media, such as Twitter and Youtube. There is support for social media to facilitate medical education by providing an open learning resource for students, enhancing learning of anatomy and providing examples of the lived experience of patients with chronic pain [57–59]. Overall evidence is however limited, particularly regarding influence on academic performance [58]. In relation to LBP, the content can additionally be problematic. For example, the most accessed videos on YouTube providing advice on LBP did not reflect guidelines recommendations and misinformation has been distributed about manual therapy [60, 61].

The influence of medical training was considered a main theme amongst students in phase 3, particularly those with no prior healthcare experience, providing further evidence for education influencing LBP beliefs. Medical training has been demonstrated to influence beliefs through brief interventions, such as 15-minute educational videos and seminars [40, 41]. Changes also occur over the course of training, with healthcare and medical students’ beliefs of chronic LBP becoming more positive, demonstrated in other research by BBQ and HC-PAIRS score changes [24, 39]. This is supported by the qualitative data, with phase 3 describing university learning opportunities such as lectures and seminars. However, the lack of change in questionnaire scores in this study may be due to the small sample size, limiting the conclusions drawn from it.

In addition, experience from clinical placements was a sub theme underlining the importance of learning in clinical environments. Discussions with patients and General Practitioners (GP) were mentioned, reflecting the large proportion of LBP consultations in primary care [62]. Patient encounters are beneficial for educating healthcare students as they may increase understanding of illness experience, improve communication and lead to more holistic care, with the latter a key approach to managing LBP [63]. Yet, as some GP’s have been shown to display fear avoidant beliefs about LBP and can give advice inconsistent with guidelines, this may be a source of conflict when students are forming beliefs about LBP. This may particularly be for those tending towards positive beliefs about LBP and who described multiple sources as influencing their beliefs [20–22, 64]. However, there were often multiple sources influencing medical students’ beliefs mentioned in this sample. Interactions with GP’s may therefore be only one source influencing students’ beliefs, in conjunction with lectures and accessing peer reviewed information such as National Institute for Health and Care Excellence (NICE) guidelines.

There are a number of limitations to this study. Firstly, the small sample size, low response rate and lack of equal representation of students in phase one limits inferential comparisons and conclusions that can be drawn in the quantitative analysis, such as comparing changes in LBP beliefs between the two-year groups using BBQ and HC-PAIRS scores. Non-response bias may account for this, as students with less exposure to LBP teaching may have less confidence about their knowledge of LBP, so are less likely to respond to the invitation [65]. Similarly, students who have not experienced LBP may have been less inclined to take part in the questionnaire, which could account for the higher prevalence of LBP amongst this sample compared with other research of medical students. The low overall response rate may also not capture the variety of attitudes amongst students, which in a larger sample may be more diverse. Additionally, whilst the questionnaires in this study have been used in research of healthcare students, they have not been validated for this specific population. Thirdly, the themes identified have limited generalisability as this sample used graduate students. Subsequent research of undergraduate students is therefore required to further evaluate medical students’ beliefs of LBP. Furthermore, no data on ethnicity was taken from participants. Future research should collect this data to ensure it is inclusive and investigates all participant characteristics.

## Conclusion

Graduate medical students in this sample tended to have positive beliefs about the outcome of LBP and functional expectations chronic LBP patients. The mean BBQ and HC-PAIRS scores are comparable to other findings of medical students. The qualitative data suggests how medical students form beliefs about the causes and management of LBP is complex. Beliefs were influenced by single or multiple sources including personal experience and medical training. Further research of medical students, including undergraduates, is required to explore these findings and understand how beliefs about LBP are influenced during medical training.

## Supplementary Information

**Additional file 1:** Sample survey.

**Additional file 2:** Supplementary index of supporting quotes.

**Additional file 3:** Raw survey data.

## Data Availability

All data generated or analysed during this study are included in this published article and its supplementary information files.

## Abbreviations

LBP: Low Back Pain
HCP: Healthcare Professional
GP: General Practitioner
BBQ: Back Beliefs Questionnaire
HC-PAIRS: Health Care Providers’ Pain and Impairment Relationship Scale
NICE: National Institute for Health and Care Excellence
BS-REC: Biomedical and Scientific Research Ethics Committee
