# Supplementary material for: What influences graduate medical students’ beliefs of lower back pain? A mixed methods cross sectional study
Source: BMC Med Educ. 2022 Aug 20;22:633. doi: 10.1186/s12909-022-03692-1 (PMC9392230; doi:10.1186/s12909-022-03692-1)
Supplement: Supplementary file 1 — Additional file 1: Sample survey. [file 12909_2022_3692_MOESM1_ESM.docx]

Additional file 1

Word document (.docx)

Title: Sample survey

Description: An example of the survey, which was formatted into ‘Qualtrics’ software and completed by participants in this study.

Sample survey

**Warwick Medical School SSC2 Survey – What do medical students believe about lower back pain and what influences their beliefs?**

The link you used to get here is anonymised and it is not able to track information identifying your responses

You can do part of the survey and come back to it another time if you wish as it will save your progress automatically. However, this facility is only possible if you return to the survey on the same internet browser on the same computer to finish the survey since the survey uses cookies to save progress.

The questionnaire will take around 10 minutes to complete.

This survey is investigating your beliefs of low back pain. Low back pain is a common condition medical students will encounter as future doctors.

If you choose to take part, this will involve filling out a short questionnaire. This will ask for basic personal information about you: your gender, age, year of study, previous university degree type, if you currently have back pain and if you have been affected by low back pain before. This will be followed questions regarding your beliefs about lower back pain.**These questions are not assessing your knowledge, there are no correct answers and are used to explore your beliefs of low back pain.**

Do I have to take part?

No. Participation in this study is completely voluntary, choosing not to take part will not affect your student grades in any way and you can refuse to take part or exit the survey before submitting at any time without penalty.

What will happen if I don’t want to carry on being part of the study?

Participation is entirely voluntary, and if you decide to withdraw from the study, it will not affect you or your educational grades in any way and you do not need to give a reason. No identifiable data will be collected from you as part of this study. You are free to withdraw at any point. If you choose to withdraw after starting the questionnaire, your responses will be saved. However, as your participation is anonymous there will be no way of identifying your responses after withdrawing.

Your consent
As described in the Participant Information Sheet, by taking part in this survey you are consenting for your data to be used in the study. All responses are anonymous. You are free to withdraw at any time by closing your browser, although you will not be able to alter your responses as they will not be saved.

Who should I contact if I want further information?
Student: John Inman [john.inman@warwick.ac.uk]
Supervisor: David Ellard [D.R.Ellard@warwick.ac.uk]

Who should I contact if I wish to make a complaint?
Any complaint about the way you have been dealt with during the study or any possible harm you might have suffered will be addressed. Please address your complaint to the person below, who is a senior University of Warwick official entirely independent of this study:

Head of Research Governance Research & Impact Services

University House

University of Warwick

Coventry

CV4 8UW

Email: researchgovernance@warwick.ac.uk

Tel: 02476 575733

If you wish to raise a complaint on how we have handled your personal data, you can contact our Data Protection Officer who will investigate the matter: DPO@warwick.ac.uk. If you are not satisfied with our response or believe we are processing your personal data in a way that is not lawful you can complain to the Information Commissioner’s Office (ICO).

For more information about this study including data usage, please refer back to the Participant Information Sheet below

[Participant information sheet](https://warwick.co1.qualtrics.com/WRQualtricsSurveyEngine/File.php?F=F_3KqopGSY7wVwBSu&download=1)

By clicking ‘I consent’:

1. I confirm that I have read and understand the information sheet for the study. I have had the opportunity to consider the information, ask questions and have had these answered satisfactorily.

2. I understand that my participation is voluntary and that I am free to withdraw at any time before submission without giving any reason, without my education being affected.

3. I understand that data collected during the study, may be looked at by individuals from The University of Warwick, where it is relevant to my taking part in this study. I give permission for these individuals to have access to my data.

4. I consent to use of anonymous questionnaire data and demographic information in the students SSC2 project. This data may also be used for future research, including impact activities following review and approval by an independent Research Ethics Committee.

5. I am happy for my data to be used in future research.

*(Choosing ‘I Consent’ takes participant to survey. Choosing ‘I Do Not Consent’ exits participant).*

Survey

1. What gender do you identify as?

- Male
- Female
- Non-Binary
- Other
- Prefer not to disclose

1. How old are you?

- 20-25
- 26-30
- 31-35
- 36-40
- 40+

1. What is your year of study?

- Phase 1
- Phase 3

1. What area of study was your previous degree or degrees in? (Open text box)

1. Are you currently experiencing lower back pain?

- Yes
- No

1. Have your previously experienced lower back pain?

- Yes
- No

What has influenced your beliefs about the causes of lower back pain? Please provide as much detail as you can

What has influenced your beliefs about the management of lower back pain? Please provide as much detail as you can

**Please indicate your general opinions about low back pain**.

Please answer all the statements and indicate if you agree or disagree with each affirmation by choosing the appropriate number on the scale. The responses range from 1 (Completely disagree) to 5 (completely agree).

|  | Completely disagree |  |  |  | Completely agree |
| --- | --- | --- | --- | --- | --- |
| 1. There is no real treatment for back pain | 1 | 2 | 3 | 4 | 5 |
| 1. Back pain will eventually stop you from working | 1 | 2 | 3 | 4 | 5 |
| 1. Back pain means periods of pain for the rest of one’s life | 1 | 2 | 3 | 4 | 5 |
| 1. Doctors cannot do anything for back pain | 1 | 2 | 3 | 4 | 5 |
| 1. Exercise is recommended for back pain | 1 | 2 | 3 | 4 | 5 |
| 1. Back pain makes everything in life worse | 1 | 2 | 3 | 4 | 5 |
| 1. Surgery is the most effective way to treat back pain | 1 | 2 | 3 | 4 | 5 |
| 1. Back pain may mean you end up in a wheelchair | 1 | 2 | 3 | 4 | 5 |
| 1. Alternative treatments are the answer to back pain | 1 | 2 | 3 | 4 | 5 |
| 1. Back pain means long periods of time off work | 1 | 2 | 3 | 4 | 5 |
| 1. Medication is the only way of relieving back pain | 1 | 2 | 3 | 4 | 5 |
| 1. Once you have had back pain there is always a weakness in the back | 1 | 2 | 3 | 4 | 5 |
| 1. Back pain must be rested | 1 | 2 | 3 | 4 | 5 |
| 1. With ageing back pain gets progressively worse | 1 | 2 | 3 | 4 | 5 |

*(Source: Symonds, T.L., Burton, A.K., Tillotson, K.M. and Main, C.J., 1996. Do attitudes and beliefs influence work loss due to low back trouble?. Occupational Medicine, 46(1), pp.25-32.)*

**Please answer the following questions regarding chronic lower back pain, defined as lower back pain persisting for more than 12 weeks.**

These questions are not assessing your knowledge and there are no correct answers and are used to explore your beliefs of low back pain.

Please answer all the statements and indicate if you agree or disagree with each affirmation by circling the appropriate number on the scale. The responses range from 1 (Completely disagree) to 7 (completely agree).

| **Chronic lower back pain = lower back pain persisting for more than 12 weeks.** | | | | | | | |
| --- | --- | --- | --- | --- | --- | --- | --- |
|  | Completely disagree |  |  |  |  |  | Completely agree |
| 1. Chronic back pain patients can still be expected to fulfil work and family responsibilities despite pain | 1 | 2 | 3 | 4 | 5 | 6 | 7 |
| 1. An increase in pain is an indicator that a chronic back pain patients should stop what he/she is doing until the pain decreases | 1 | 2 | 3 | 4 | 5 | 6 | 7 |
| 1. Chronic back pain patients cannot go back to normal life activities when they are in pain | 1 | 2 | 3 | 4 | 5 | 6 | 7 |
| 1. If their pain would go away, chronic back pain patients would be every bit as active as they used to be | 1 | 2 | 3 | 4 | 5 | 6 | 7 |
| 1. Chronic back pain patients should have the same benefits as disabled people because of their chronic pain problem | 1 | 2 | 3 | 4 | 5 | 6 | 7 |
| 1. Chronic back pain patients owe it to themselves and those around them to perform their usual activities even when their pain is bad | 1 | 2 | 3 | 4 | 5 | 6 | 7 |
| 1. Most people expect too much of chronic back pain patients, given their pain | 1 | 2 | 3 | 4 | 5 | 6 | 7 |
| 1. Chronic back pain patients have to be careful not to do anything that might make their pain worse | 1 | 2 | 3 | 4 | 5 | 6 | 7 |
| 1. As long as they are in pain, chronic back pain patients will never be able to live as well as they did | 1 | 2 | 3 | 4 | 5 | 6 | 7 |
| 1. When their pain gets worse, chronic back pain patients find it very hard to concentrate on anything | 1 | 2 | 3 | 4 | 5 | 6 | 7 |
| 1. Chronic back pain patients have to accept that they are disabled persons due to their chronic pain | 1 | 2 | 3 | 4 | 5 | 6 | 7 |
| 1. There is no way that chronic back pain patients can return to doing the things that they used to do unless they first find a cure for their pain | 1 | 2 | 3 | 4 | 5 | 6 | 7 |
| 1. Chronic back pain patients find themselves frequently thinking about their pain and what it has done to their life | 1 | 2 | 3 | 4 | 5 | 6 | 7 |
| 1. Even though their pain is always there, chronic back pain patients often don’t notice it at all when they are keeping themselves busy | 1 | 2 | 3 | 4 | 5 | 6 | 7 |
| 1. All of chronic back pain patients problems would be solved if their pain would go away | 1 | 2 | 3 | 4 | 5 | 6 | 7 |
|  | | | | | | | |

*(Source: Rainville J, Bagnall D, Phalen L. Health care providers’ attitudes and beliefs about functional impairments and chronic back pain. Clin J Pain. 1995;11(4): 287–95. PubMed PMID: 8788576; eng)*

**Thanks for completing this questionnaire. Your response has been recorded**
