# Supplementary material for: What influences graduate medical students’ beliefs of lower back pain? A mixed methods cross sectional study
Source: BMC Med Educ. 2022 Aug 20;22:633. doi: 10.1186/s12909-022-03692-1 (PMC9392230; doi:10.1186/s12909-022-03692-1)
Supplement: Supplementary file 2 — Additional file 2: Supplementary index of supporting quotes. [file 12909_2022_3692_MOESM2_ESM.docx]

Additional file 2

Word document (.docx)

Title: Supplementary index of supporting quotes

Description: Further illustrative quotes for supporting the themes described in this study.

| Supplementary index of supporting quotes | |
| --- | --- |
| Theme: Source of influence | |
| Sub-theme | Quote [Participant ID] |
| Single source | “My own experience with back pain.. going through my GP, for scans and physio etc” [1.5] |
|  | “Work, work, work. Physio colleagues” [1.7] |
|  | “My experience of lower back pain and how it affected my work life and hobbies” [2.5] |
|  | “Seeing family members with lower back pain” [2.6] |
|  | “Clinical placements where I’ve had the opportunity to explore different causes in histories and examination” [3.4] |
|  | “I believe my own lower back pain is caused by previous strain from poor manual handling on my part when I used to work as a Health care assistant. More generally I am unsure on the mechanisms of lower back pain” [3.5] |
|  | “My own experience with sports injuries causing back pain” [3.8] |
|  | “Personal experience” [4.9] |
|  | “Experience helping family members with severe lower back pain “[6.2] |
| Multiple sources | “Guidelines, lecture content, contact with patients” [2.1] |
|  | “Previous episodes and how I managed it myself. Influenced by beliefs as a physiotherapist and by my colleagues Influence of vocal people on social media in physio world” [2.3] |
|  | “Previous physiotherapy, consultations with doctors, information on the internet” [2.9] |
|  | “Medical school teaching Own methods Patients experience” [3.1] |
|  | “NICE guidelines and clinical placements” [3.4] |
|  | “Lectures at university, GP placement” [3.8] |
|  | “Med school education, physiotherapists, chiropractor, GP, family, friends” [3.9] |
|  | “Personal experience and that of friends and family (anecdotal) Phase 2 lectures on Lower Back Pain and MSK drugs GP Placement - seeing patients with the GPs and first contact physio” [4.0] |
|  | “Conversations with family members regarding their pain as well as the lectures provided my the medical school” [4.7] |
|  | “Personal experience (due to sports), seeing patients at GP, stories from friends, family members, colleagues”\| [5.1] |
|  | “What friends/family/social media says. Eg. having bad posture can cause lower back pain” [5.3] |
| Theme: Influence of personal experience | |
| Sub-theme | Quote [Participant ID] |
| Individual’s personal experience with LBP | “My own experience with back pain.. going through my GP, for scans and physio etc” [1.5] |
|  | “My experience of lower back pain and how it affected my work life and hobbies” [2.5] |
|  | “Personal experience - I have got lower back pain from driving long distances from work and sitting down a lot from work” [3.2] |
|  | “I believe my own lower back pain is caused by previous strain from poor manual handling on my part when I used to work as a Health care assistant”  In my personal experience I have had osteopathy on my lower back and found that to help, as well as stretches and exercises.” [3.5] |
|  | “My own experience with sports injuries causing back pain” [3.8] |
|  | “Personal experience and that of friends and family (anecdotal) Phase 2 lectures on Lower Back Pain and MSK drugs GP Placement - seeing patients with the GPs and first contact physio” [4] |
|  | “My own experience.. has informed my beliefs about lower back pain including causes and how debilitating they can be.” [4.3] |
|  | “Personal experience” [4.9] |
|  | “I try not to use my personal experience too much as I believe that my experience is specific to my case and it would not be appropriate to use the course of my back pain as a template for how back pain will affect everyone” [4.6] |
|  | “Exercising and general fitness have had a huge impact in improving my back pain” [5.2] |
|  | “Own experience” [5.4] |
| Personal experience of others with LBP | “Hearing people who live with chronic pain's experiences of pain being dismissed due to weight and their age” [1.4] |
|  | “Others experiences such as family members who experience sciatica and have lower back pain. “[1.8] |
|  | “My own. My Dads.”  “I think my dad takes co-codramol.” [1.9] |
|  | “Family experiences, where medication hasn't helped.” [2.4] |
|  | “Seeing family members with lower back pain” [2.6] |
|  | “Family history- my mother suffered with back pain. I have memories of her unable to move and lying on the floor, believing that helped. She often stated that her work and lifting caused the pain” [2.8] |
|  | “Family/friends experiences - relatives have had spondylolysis, friends have ankylosing spondylitis and others have had slipped discs. One of my friends has managed their slipped discs with just physio, the ones with ankylosing spondylitis and spondylolysis have needed lot of pain meds and other meds to be functional. Relatives with muscular back pain have had massages which they find helpful. Something that has really stuck with me was talking to a patient with a long term back issue who said they regret getting back surgery for it and would advice people to avoid it at all costs as the side effects of neuro issues are likely and not nice to live with” [3.2] |
|  | “Information from friends and family” [3.5] |
|  | “Discussion with patients in work as pharmacist” [3.3] |
|  | “Family experience of lower back pain generally being due to the family member being quite unfit and unhealthy” [3.8] |
|  | “Personal experience and that of friends and family (anecdotal)” [4] |
|  | “meeting with patients with low back pain” [4.6] |
|  | “Conversations with family members regarding their pain” [4.7] |
|  | “Personal experience and experience of family members. For example, my family members benefit from the use of a chiropractor whereas it provided no benefit for me.” [4.9] |
|  | “seeing patients at GP, stories from friends, family members” [5.1] |
|  | “Experience helping family members with severe lower back pain” [6.2] |
|  | “People I have seen with low back pain. People who I know have really struggled, but have got better eventually with physio, as long as they persevere at the exercises”  “The experiences with people I know who have had lower back pain. For these people sustained physio ecpercies worked in the long term, but not so much in the short term.” [6.5] |
| Clinical personal experience of LBP | “Knowledge & experience of supporting patients as a physiotherapist both with acute & chronic lower back pain” [1.1] |
|  | “Studying physiotherapy has given me a much better insight in back pain management” [1.3] |
|  | “Worked as physiotherapy assistant and helped patients with back pain” [1.6] |
|  | “I also had exposure assessing many lower back pain patients and seeing the different causes” [2.1] |
|  | “Influenced by beliefs as a physiotherapist” [2.3] |
|  | “My experience of nursing is that lower back pain is caused by trauma, mechanical and tear and osteoporosis. I’ve also seen it being exacerbated by sedentary lifestyles and patients who are overweight.” [3] |
|  | “Discussion with patients in work as pharmacist” [3.3] |
|  | “As a previous carer and healthcare worker I know moving patients and personal cares can cause it” [6.3] |
| Theme: Influence of medical education | |
| Sub-theme | Quote [Participant ID] |
| From university | “Family experiences, where medication hasn't helped. This was backed up by a medical school lecture that argued that medication normally isn't useful.” [2.4] |
|  | “We had a lecture last year which indicated that analgesia was ineffective in management of lower back pain, and interventions such as those for chronic pain were more effective” [3.3] |
|  | “I think both the formal education of management throughout Phase 2 in particularly has added more knowledge.” [3.7] |
|  | “Lectures at university” [3.8] |
|  | “Lectures that we have received on the different causes of low back pain and the fluctuating nature of MSK low back pain.”  “Lectures from experts/those who have researched this topic and have a good understanding of the research base. Discussions with GPs and patients, although I try to put these in the context of what we're taught in lectures. The understanding of anatomy and correct posture that we have been taught during Phase I.” [4.6} |
|  | “The medical school lectures that focus on the pharmacological and non-pharmacological pain relief for lower back pain” [4.7] |
|  | “Formal teaching,” [5.1] |
|  | “I think lectures on the management of lower back pain in Phase II have influenced my beliefs the most” [5.3] |
|  | “Lectures and ADs (*Academic day)* in phase II based on back pain and how to treat the condition. A lecturer on our course explained how difficult lower back pain can be to manage due to the interplay between physical and psychological components.” [5.5] |
|  | “Phase 2 lectures - we were taught that it's a bio-psycho-social issue, not just a purely biological/mechanical one” [5.6] |
|  | “teaching in medical school” [5.9] |
|  | “CBL *[Case based learning]* case on how lower back pain is linked with chronic conditions such as depression” [6] |
|  | “The teachings on my course” [6.2] |
|  | “learning through lectures” [6.3] |
| From self-study | “Personal research on the internet for the most part” [2.2] |
|  | “Information on the internet” [2.9] |
|  | “Learning more about lower back pain, from other professionals and through reading information on the topic” [4.4] |
|  | “remaining knowledge I would have got from studying, why for me mainly comprises of online resources - Zeros to Finals, YouTube videos.” [4.8] |
|  | “NHS website, medicine teaching” [5.4] |
|  | “I also found reading the BMJ best practice useful” [5.7] |
|  | “Google/NHS website” [5.9] |
|  | “learning through lectures/bmj best practice and NICE guidelines” [6.3] |
| From clinical placement | “Clinical placements where I've had the opportunity to explore different causes in histories and examinations.” [3.4] |
|  | “GP Placement - seeing patients with the GPs and first contact physio” [4] |
|  | “Experience during GP placements resulted in many presentations of back pain, including lower back pain.”  “Once again, GP placements - more often than not, lower back pain seems to be a chronic issue without any confirmed aetiology. Management in this case is usually analgesia.” [4.3] |
|  | “Discussions with GPs when in their practice and meeting with patients with low back pain.” [4.6] |
|  | “Lower back pain is a common presenting complaint at GP - during GP days I saw some patients with lower back pain.”  “Mainly my time during GP placements, this is where I got direct hands on knowledge on treatment and management (Pain management and imaging to exclude insidious causes),” [4.8] |
|  | “seeing patients at GP” [5.1] |
|  | “Going on GP placements and seeing patients with lower back pain has also reinforced these beliefs.”  “From our Phase 2 lectures and also from seeing patients in GP placements, it seems like it's a balance of pain medication, and perhaps physiotherapy exercises, with not much else of use.” [5.6] |
|  | “Learning through gp placements about the causes of back pain, such as mechanical and other causes such as depression  I found GP placements to be the most useful when learning about low back pain as it is a common presenting complaint. I also found reading the BMJ best practice useful” [5.7] |
|  | “The course - in particular GP and ED” [6.1] |
|  | “being in GP practice” [6.3] |
| Minor themes | |
|  | Quote [Participant ID] |
| Complexity with LBP | “The management seems more about relieving the symptoms/pain than progressive improvement.” [1.9] |
|  | “but my perception is these interventions are not effective for everyone and many people simply live with near constant lower back pain which is exacerbated at times.” [3] |
|  | “more often than not, lower back pain seems to be a chronic issue without any confirmed aetiology.” [4.3] |
|  | “it seems like it's a balance of pain medication, and perhaps physiotherapy exercises, with not much else of use.” [5.6] |
|  | “Hard to treat - depends on type of pain/diagnosis” [6] |
|  | “Difficult to treat but very common” [6.1} |
|  | “I have realised medication is not always the best cure of even first line treatment, but physio/exercise can be more beneficial watch and wait approach” [6.3] |
| Influence of previous degree | “Knowledge & experience of supporting patients as a physiotherapist both with acute & chronic lower back pain”  *“*Knowledge, experience and exposure as a physiotherapist” [1.1] |
|  | “previous degree knowledge of anatomy” [1.6] |
|  | “Previous undergraduate education in osteopathy.. . I also had exposure to assessing many lower back pain patients and seeing the different causes [2.1 |
|  | “Previous degree as a physiotherapist”[2.3] |
|  | “the basis for most of my understanding about different types of pain management has stemmed from my Neuropsychiatry studies” [3.7] |
| Influence of social media | “Information found on social media, particularly regarding strength training (not necessarily from qualified or trained individuals)” [1.3] |
|  | “When I personally get lower back pain, e.g during my period, has influenced what I suspect causes may be as well as social media awareness on correct exercise form to prevent lower back injury.”  Mostly the treatment options that work for me e.g heat and massage tend to relieve lower back pain for me. Other things include social media professionals such as physiotherapists and chiropractors who show exercises to help manage lower back pain. [1.8] |
|  | “Other things include social media professionals such as physiotherapists and chiropractors who show exercises to help manage lower back pain.. Influence of vocal people on social media in physio world.” [2.3] |
|  | the remaining knowledge I would have got from studying, why for me mainly comprises of online resources - Zeros to Finals, YouTube videos. [4.8] |
|  | What friends/family/social media says. Eg. having bad posture can cause lower back pain. [5.4] |
